# Supplementary material for: Targeting the IRE1α-XBP1 signaling axis impairs tumor growth and promotes myogenic differentiation in rhabdomyosarcoma
Source: Commun Biol. 2026 May 6;9:937. doi: 10.1038/s42003-026-10184-1 (PMC13351075; doi:10.1038/s42003-026-10184-1)
Supplement: Supplementary file 2 — Supplementary Information [file 42003_2026_10184_MOESM2_ESM.pdf]

Supplementary Information File

Targeting the IRE1α-XBP1 signaling axis impairs tumor growth and promotes myogenic differentiation in rhabdomyosarcoma

Anh Tuan Vuong, Aniket S Joshi, Phuong T. Ho, Meiricris Tomaz da Silva, Bin Guo, Meghana V. Trivedi, and Ashok Kumar

This file contains **Figures S1-S7** and **Tables S1 and S2.**

Supplemental Figures and Legends

FIGURE S1

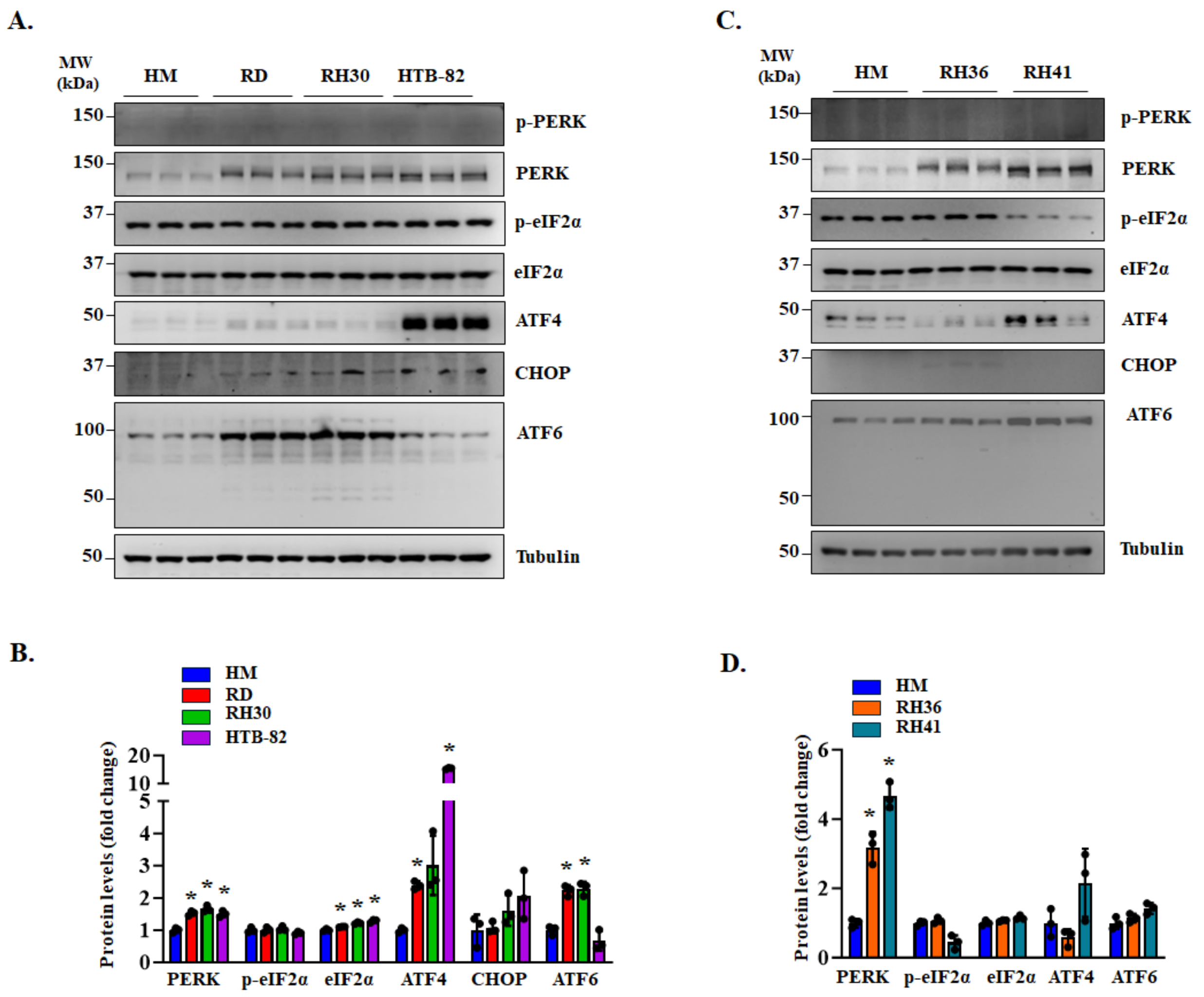

FIGURE S1. Expression of ER stress/UPR markers in RMS cell lines. (A, C) Immunoblots and **(B, D)** densitometry analysis showing the levels of phosphorylated PERK (p-PERK), total PERK, p-eIF2α, total eIF2α, ATF4, CHOP, ATF6 and Tubulin protein in human myoblasts (HM), and RD, RH30, HTB-82, RH36, and RH41 cell lines. n=3 biological replicates per group. Data are presented as mean ± SD. *p<0.05, values significantly different from HM analyzed by unpaired Student *t* test.

FIGURE S2

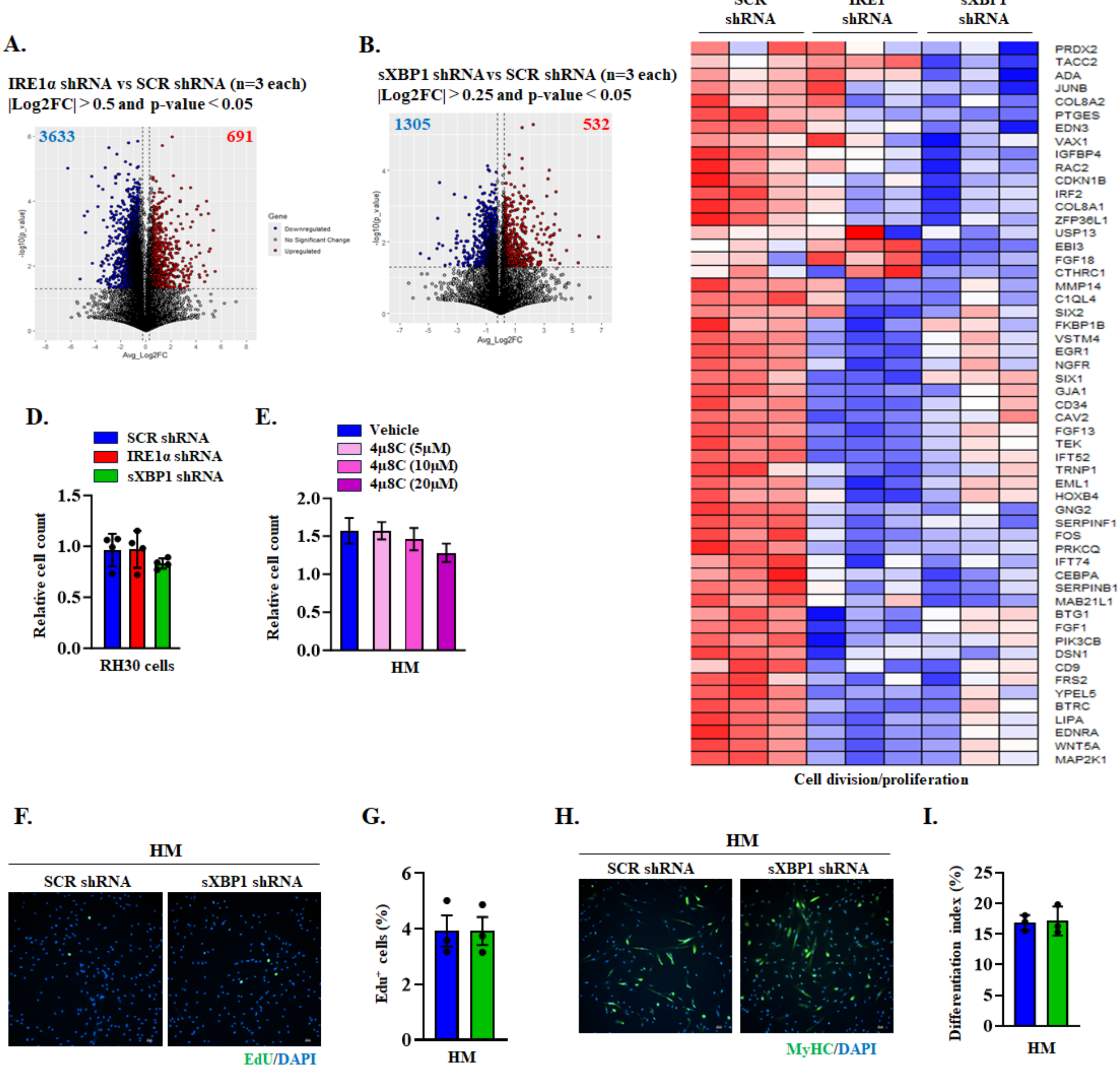

FIGURE S2. Effect of knockdown of IRE1α or sXBP1 on proliferation of RMS cells. Volcano plots showing differentially expressed genes in **(A)** IRE1α shRNA and **(B)** sXBP1 shRNA expressing RD cells compared with SCR shRNA expressing RD cultures. **(C)** Heatmap generated after analysis of RNA-seq dataset show relative mRNA levels of various molecules involved in cell division and proliferation-related genes in RD cultures expressing scrambled (SCR), IRE1α, or sXBP1 shRNA. **(D)** Relative number of RH30 cells expressing SCR, IRE1α, or sXBP1 shRNA after culturing for 72 h in growth medium. **(E)** Relative number of human myoblasts (HM) after treatment with indicated concentrations of 4μ8C for 4 days in growth medium. **(F)** Representative images of control and sXBP1 knockdown HM after performing EdU staining. Scale bar, 100 μm. **(G)** Quantification of percentage of EdU^{+} cells in control and sXBP1 knockdown HM cultures. **(H)** Representative images of control and sXBP1 knockdown HM after performing immunostaining for MyHC. Scale bar, 100 μm. **(I)** Quantification of

differentiation index in control and sXBP1 knockdown HM cultures. n=3 biological replicates per group. Data are presented as mean ± SD. No significant difference was observed using unpaired Student t test.

FIGURE S3

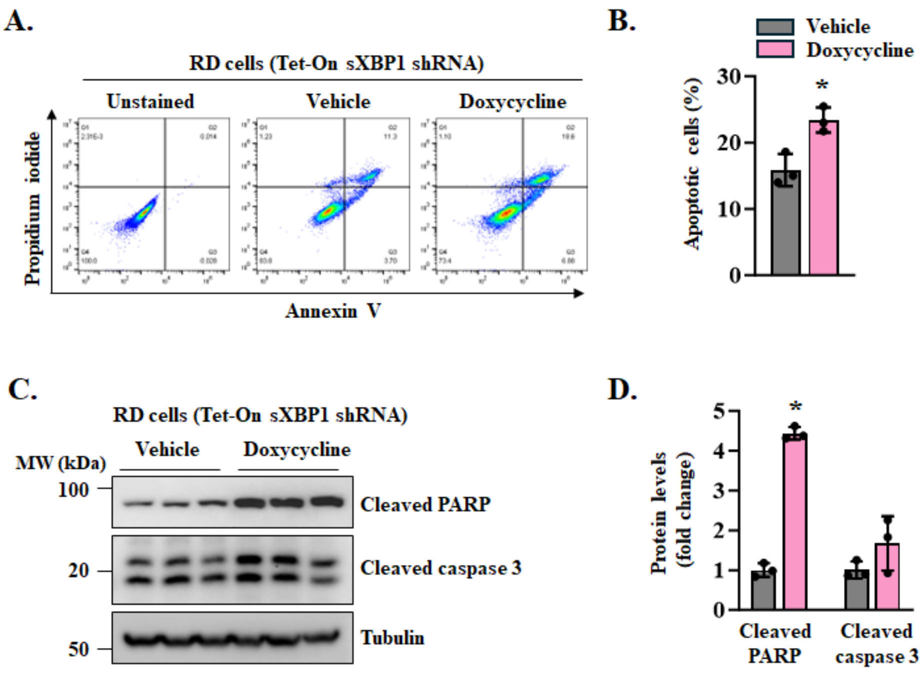

FIGURE S3. Inducible knockdown of XBP1 promotes apoptosis in RD cells. RD cells stably expressing Tet-On XBP1 shRNA were treated with vehicle alone or 1µg/ml doxycycline for 5days followed by propidium iodide (PI) and Annexin V staining and FACS analysis. **(A)** Scatter plots and **(B)** quantification of proportion of apoptotic cells in control and doxycycline-treated RD cultures. n=3 biological replicates per group. Data are presented as mean ± SD. *p<0.05, values significantly different from vehicle-treated control cultures analyzed by unpaired Student *t* test. **(C)** Immunoblots and **(D)** densitometry analysis showing levels of cleaved PARP and cleaved Caspase-3 protein in control and doxycycline-treated RD cultures. n=3 biological replicates per group. Data are presented as mean ± SD. *p<0.05, values significantly different from vehicle-treated control cultures analyzed by unpaired Student *t* test.

FIGURE S4

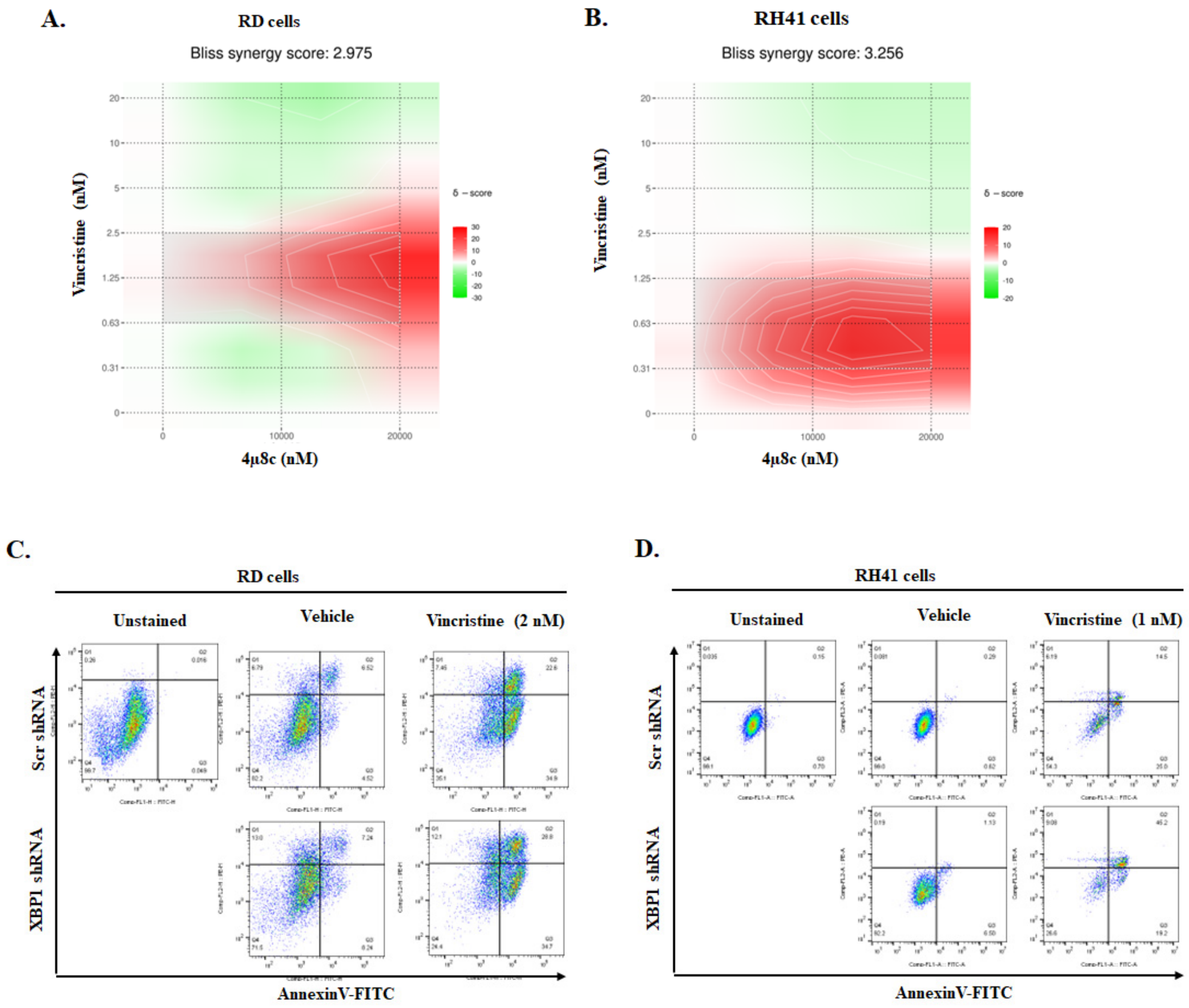

FIGURE S4. Inhibition of XBP1 increases vincristine-induced apoptosis in RMS cells. Vincristine and 4μ8C interaction analysis was performed using the Bliss independence model, and synergy scores were calculated using SynergyFinder. Drug combination matrix showing the effects of varying concentrations of vincristine and 4μ8C on the viability of (A) RD cells, **(B)** RH41 cells. Positive values of Bliss scores suggest synergistic interactions between vincristine and 4μ8C. Scatter plots of FACS analysis presented here demonstrate that knockdown of XBP1 increases the vincristine-induced apoptosis in **(C)** RD cells and **(D)** RH41 cells. Quantification is provided in main Figure 4F.

FIGURE S5

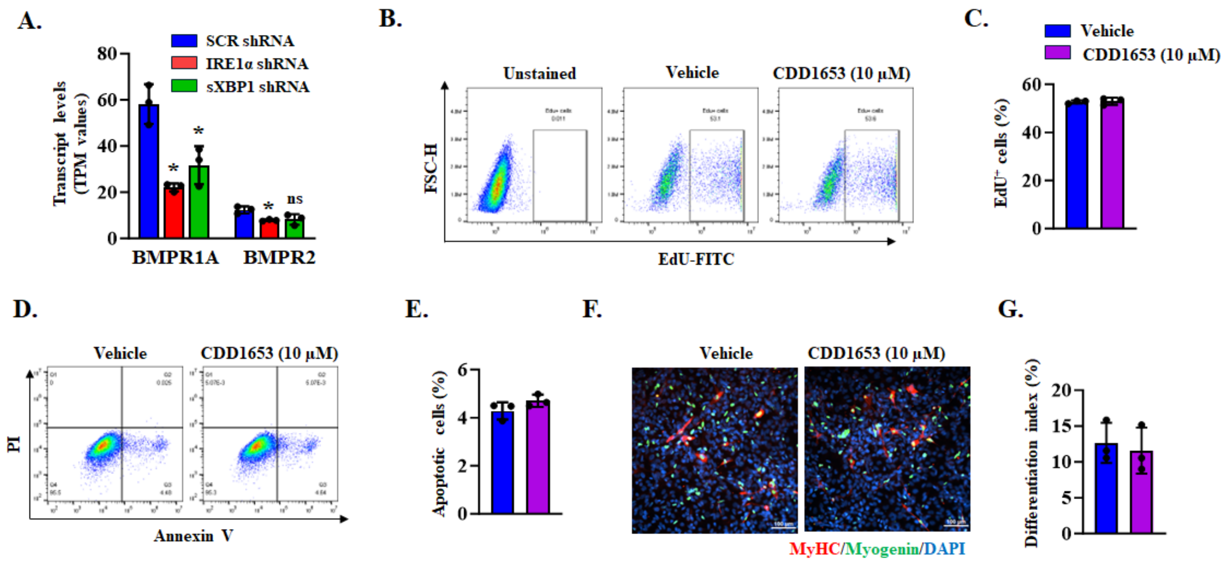

FIGURE 5. Effect of BMPR2 inhibition on RD cells. (A) TPM values from RNA-seq dataset for the expression of BMPR1A and BMPR2 in RD cells expressing scrambled (SCR), IRE1α, or sXBP1 shRNA. n=3 biological replicates per group. Data are presented as mean ± SD. *$p < 0.05$, values significant different from corresponding SCR shRNA expressing RD cultures. **(B)** Scatter plots of flow cytometry, and **(C)** quantification of EdU^+ cells in cultures treated with vehicle alone or 10µM CDD1653. **(D)** Scatter plots, and **(E)** quantification of Annexin V^+ cells in cultures treated with vehicle alone or 10µM CDD1653. **(F)** Representative images of vehicle and CDD1653-treated RD culture after immunostaining for myogenin and MyHC proteins. Nuclei were stained with DAPI. **(G)** Quantification of differentiation of index in control and CD1653-treated RD cultures. n=3 biological replicates per group. Data are presented as mean ± SD. No significant difference was observed using unpaired Student *t* test.

FIGURE S6

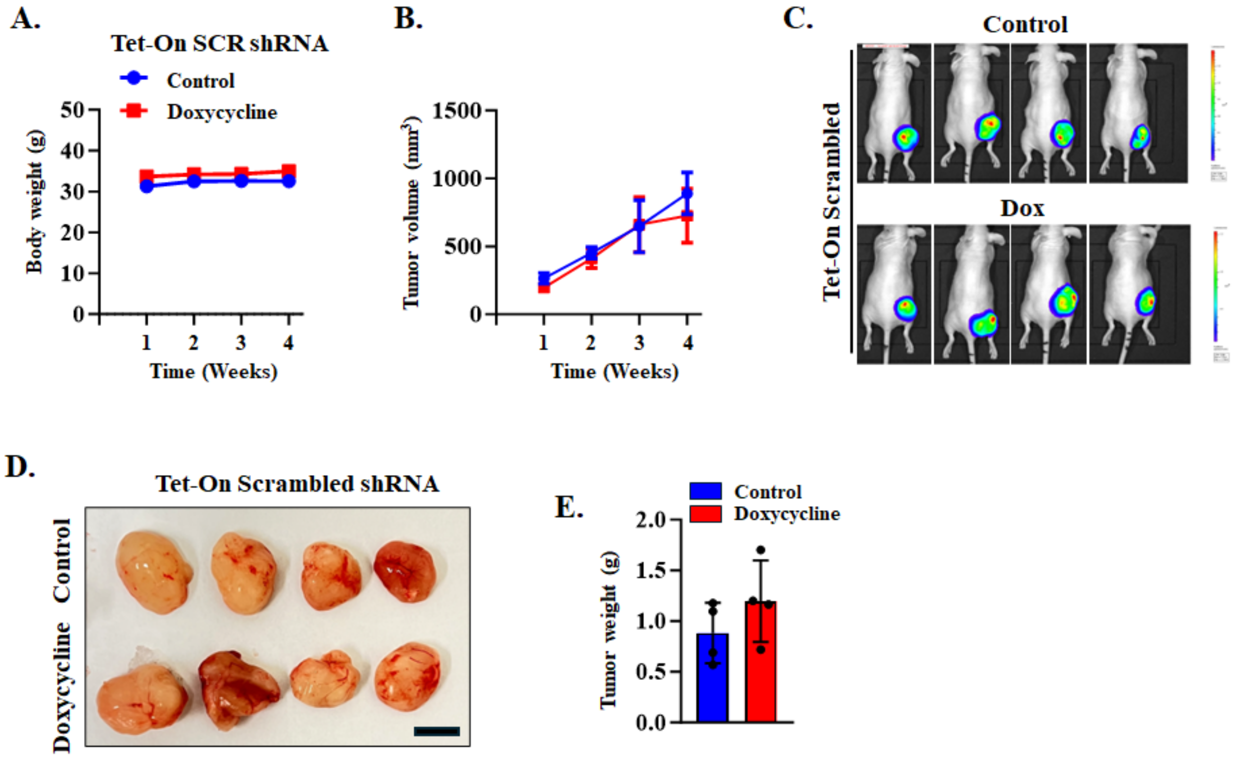

FIGURE S6. Treatment with doxycycline alone does not alter tumor growth in RD xenografts. (A) Body weight of mice inoculated with Tet-On scrambled (SCR) shRNA expressing RD cells fed with normal chow or doxycycline-containing chow. **(B)** Average tumor volume in mice fed with normal chow or doxycycline containing chow. n=4 mice in each group. Data are presented as mean ± SD. No significant difference was observed using unpaired Student *t* test. **(C)** Bioluminescence imaging showing presence RD xenograft in control and doxycycline-treated nude mice. **(D)** Images of tumors at the time of euthanizing the mice between control and doxycycline-treated groups. Scale bar, 1cm. **(E)** Quantification of wet weight of tumors in control and doxycycline-treated mice. n=4 mice per group. Data are presented as mean ± SD. No significant difference was observed using unpaired Student *t* test.

FIGURE S7

Figure 1D

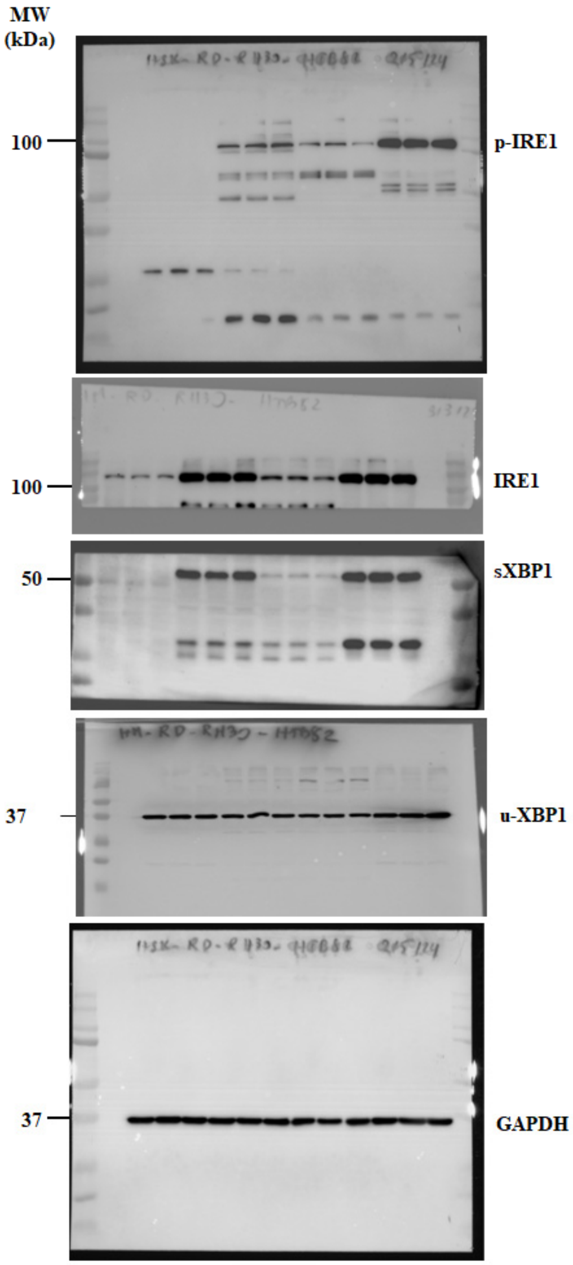

Figure 1F

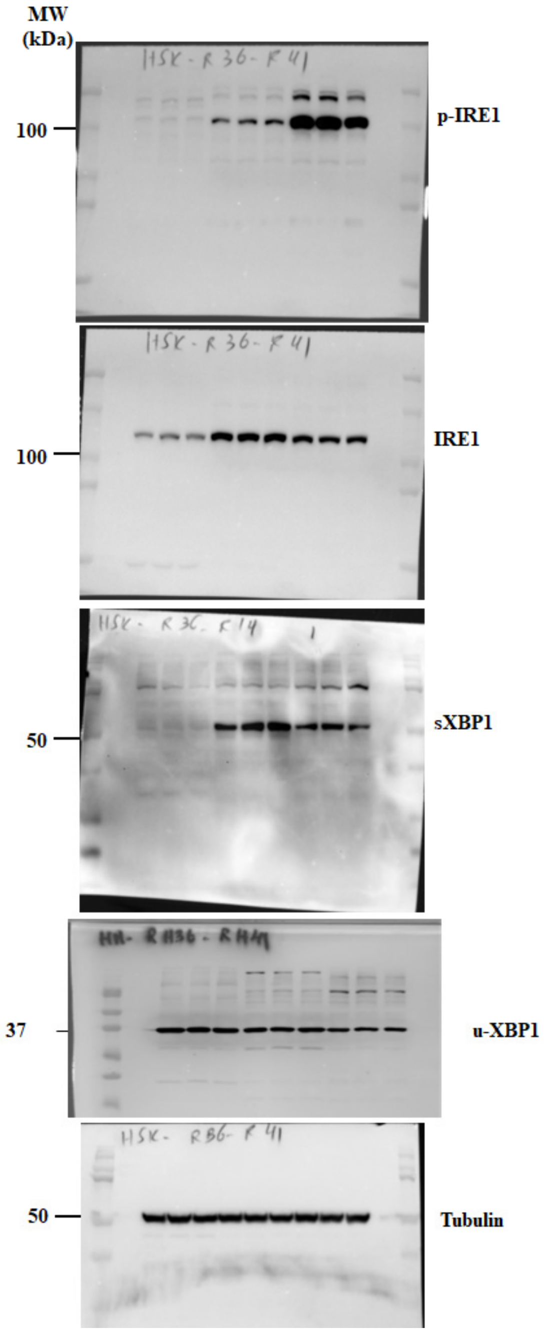

FIGURE S7 (continuation)

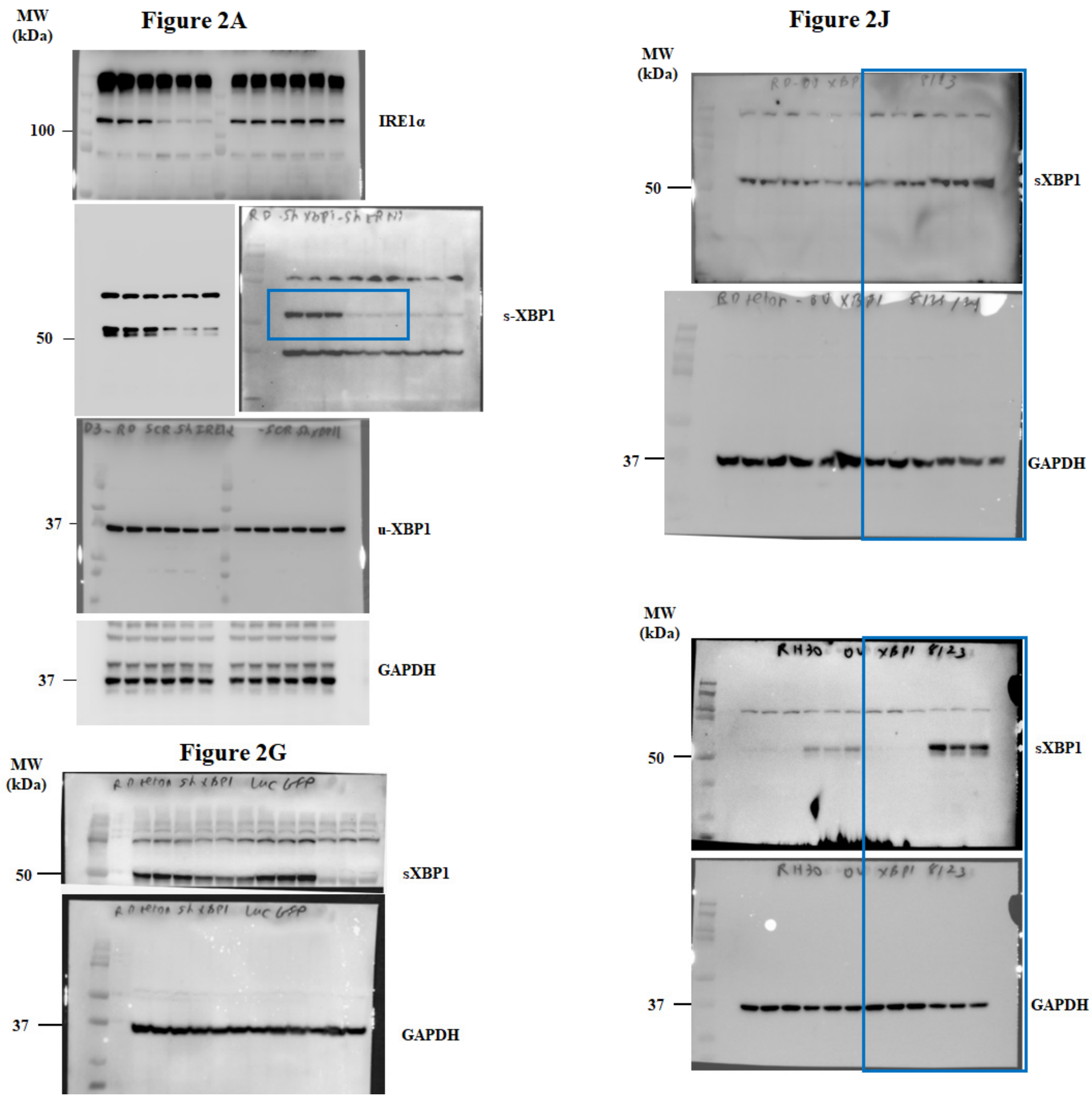

FIGURE S7 (continuation)

Figure 3C

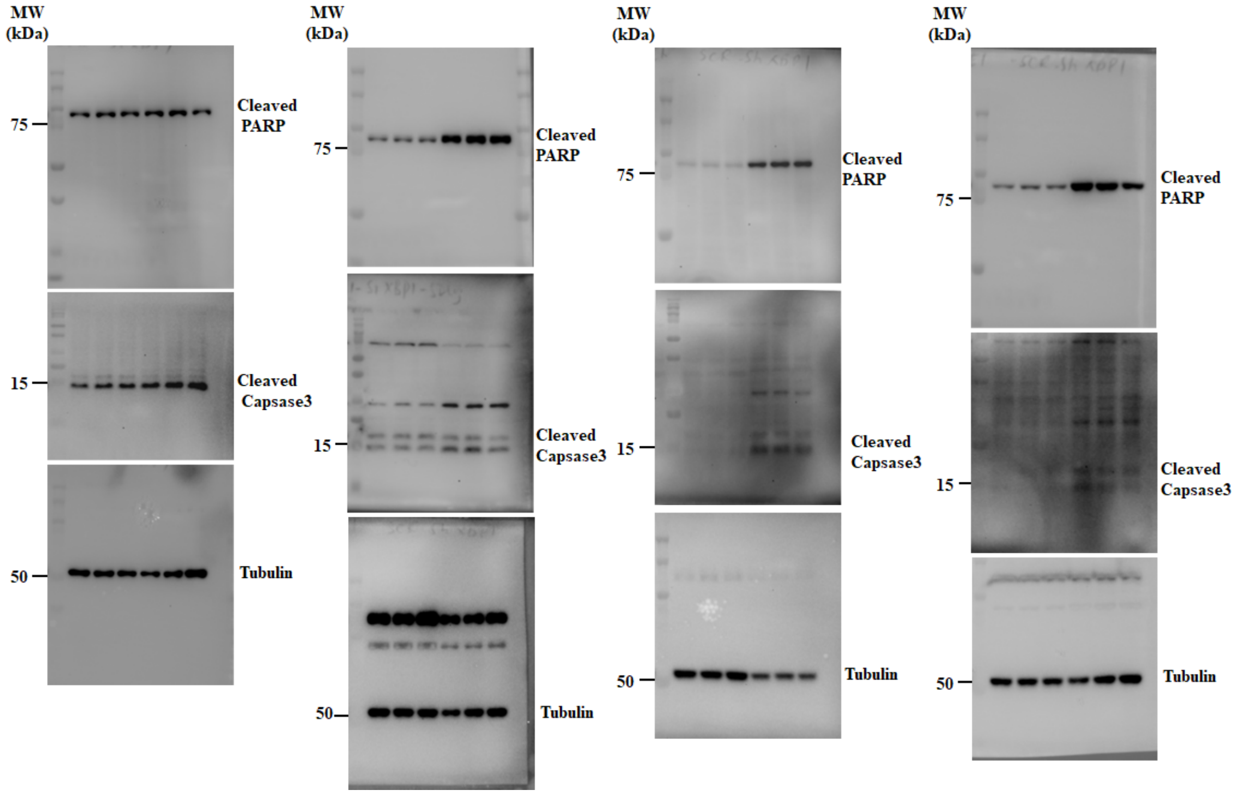

FIGURE S7 (continuation)

Figure 3G

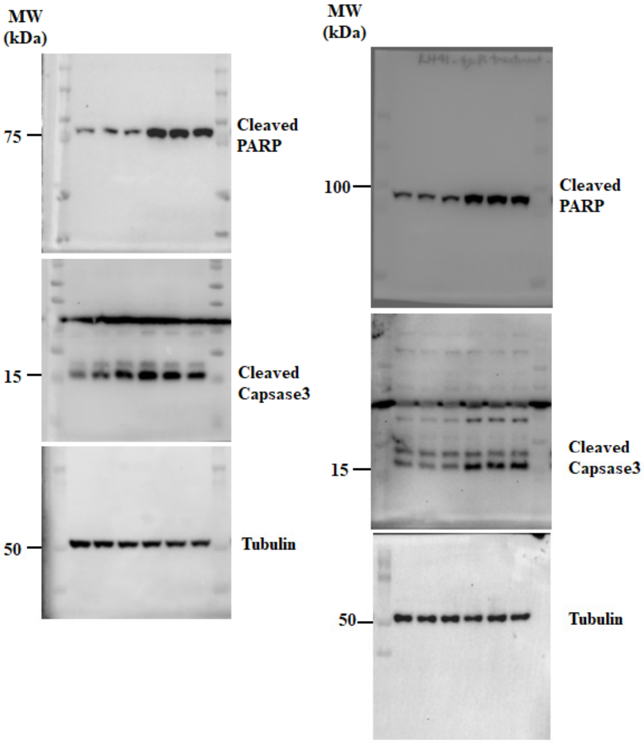

Figure 5B

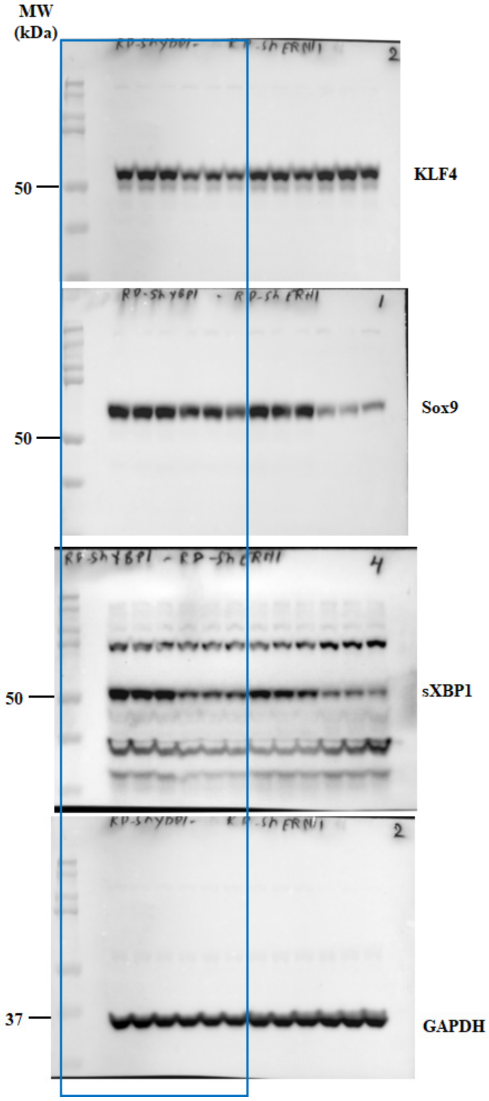

FIGURE S7 (continuation)

Figure 6L

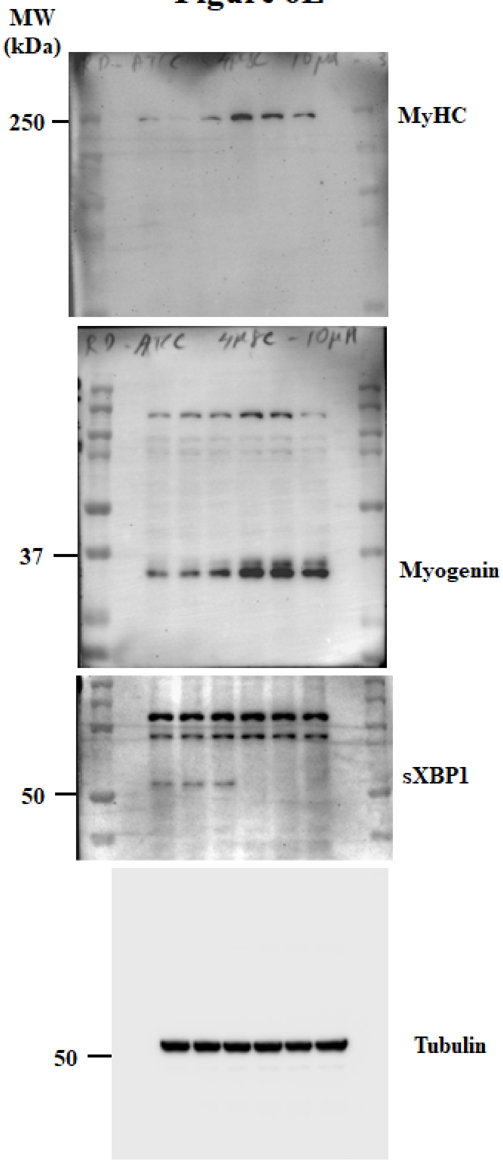

FIGURE S7 (continuation)

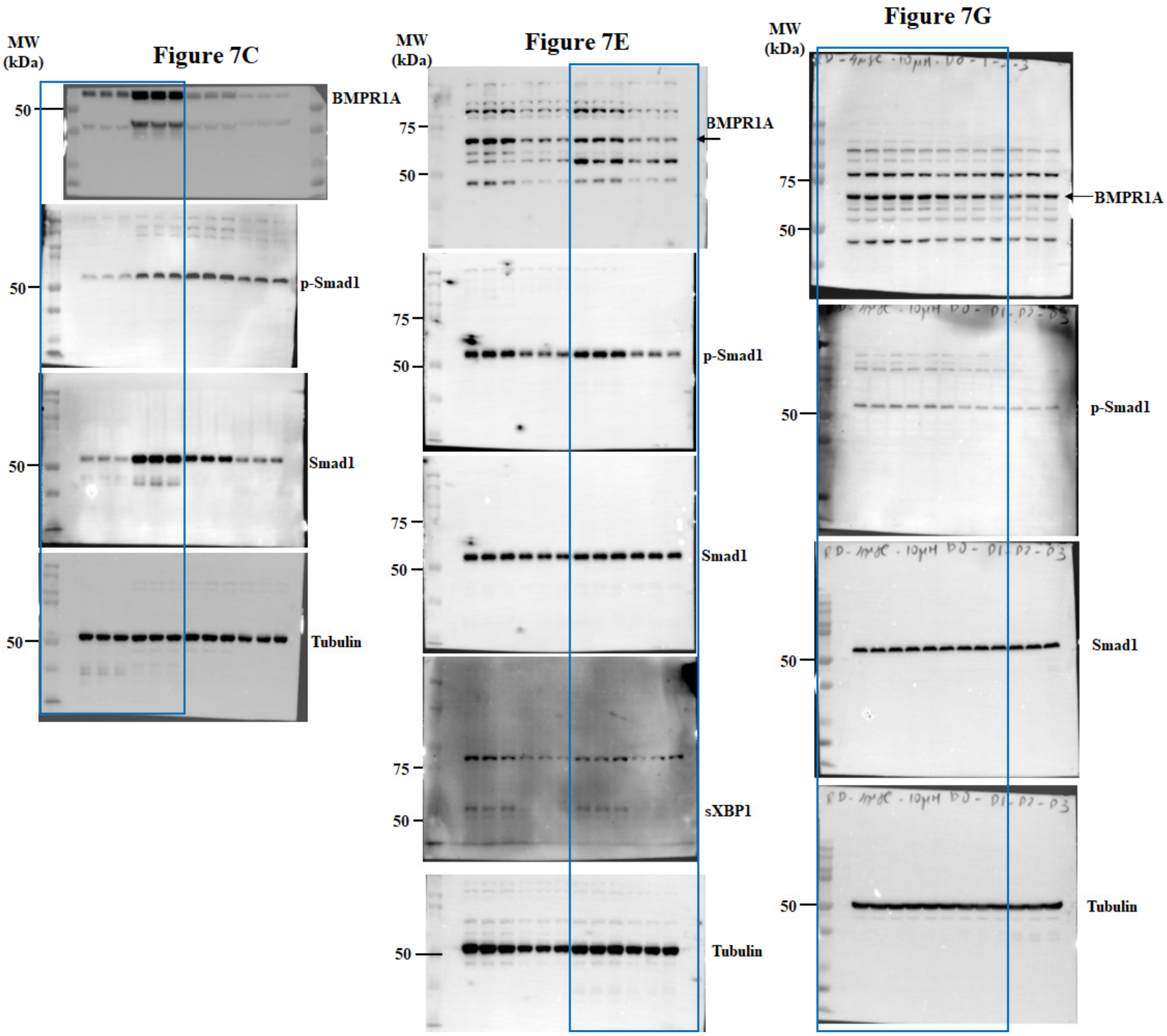

FIGURE S7 (continuation)

Figure 7I

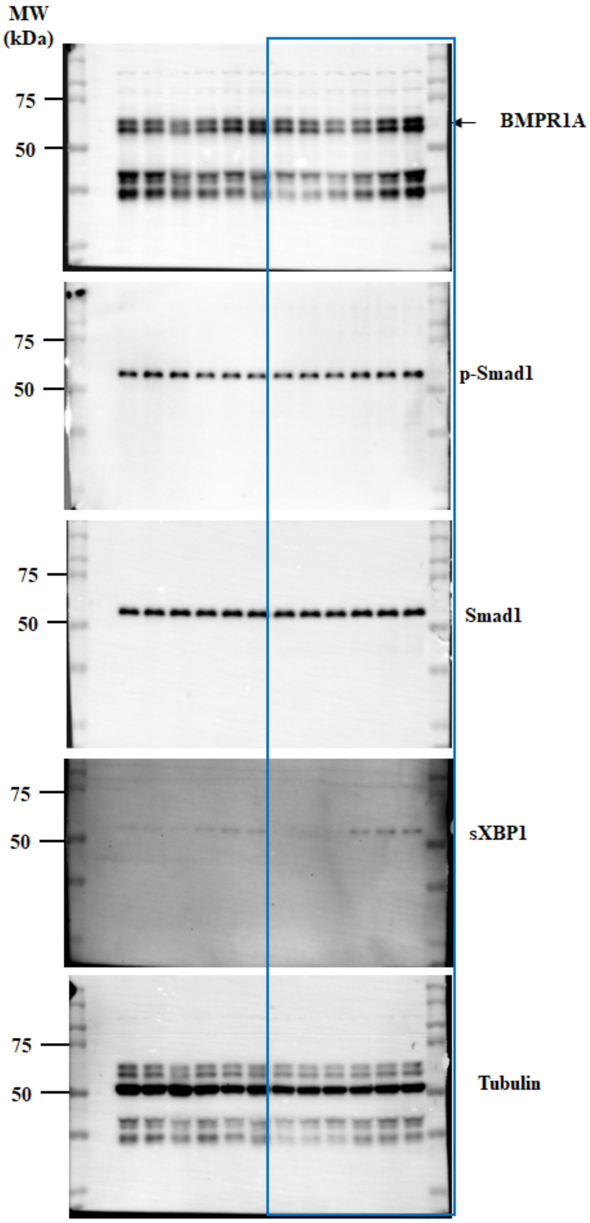

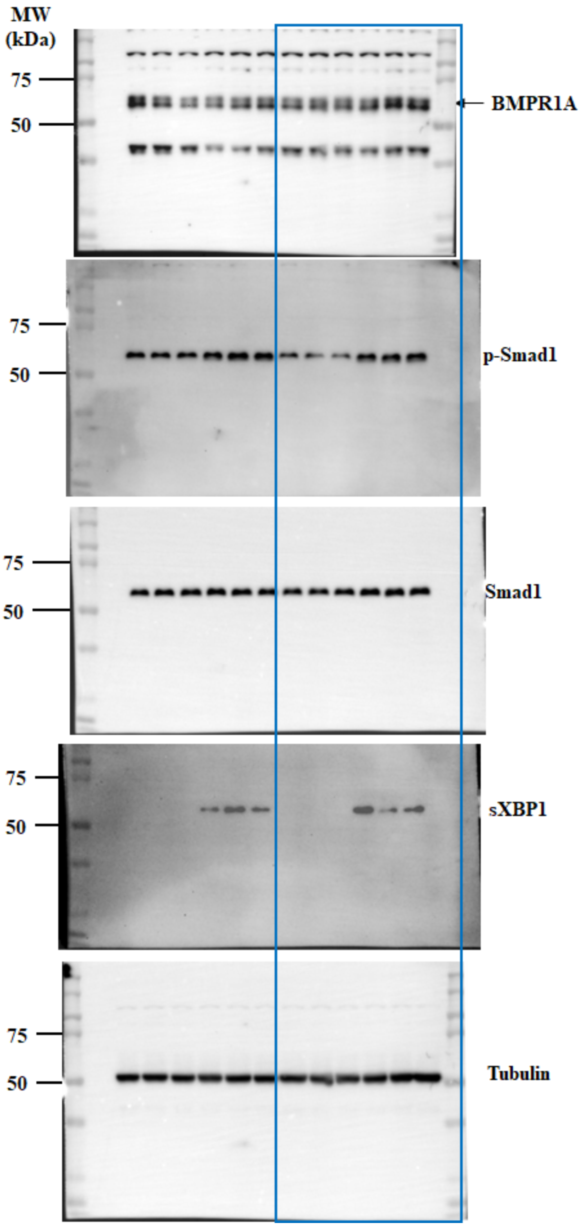

FIGURE S7 (continuation)

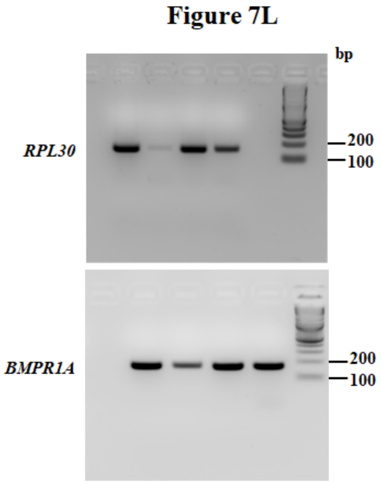

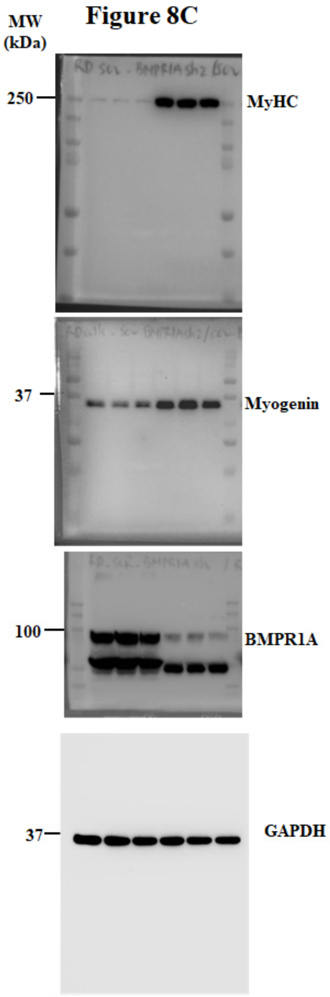

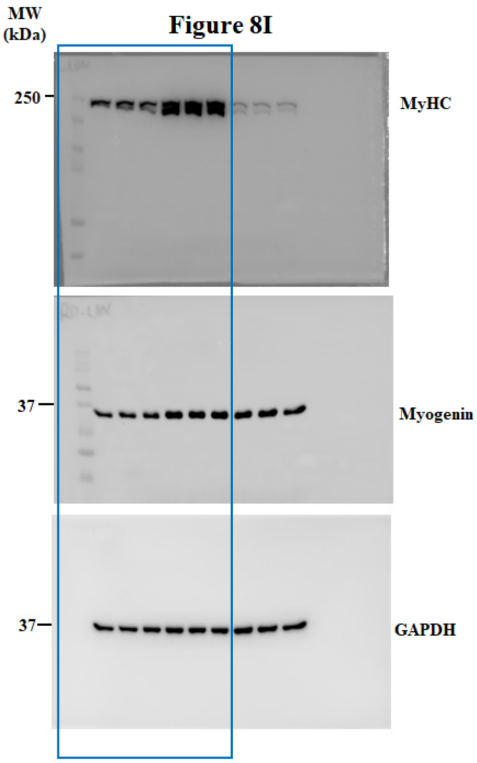

FIGURE S7 (continuation)

Figure 9K

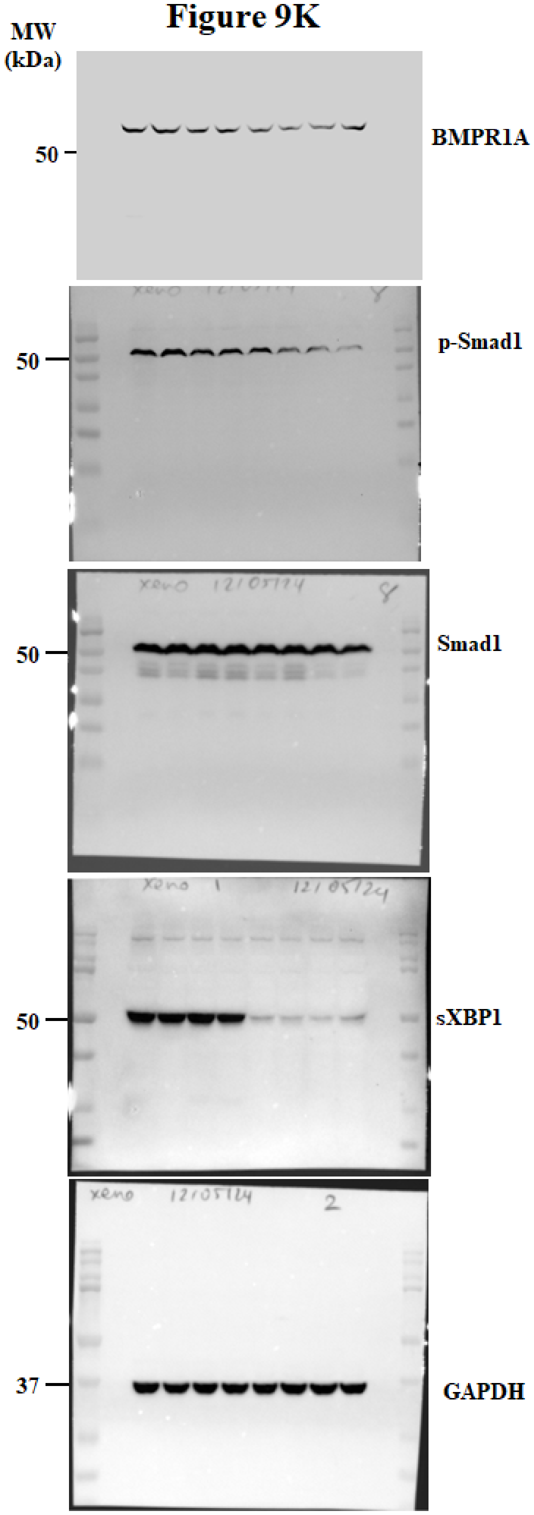

Figure S1A

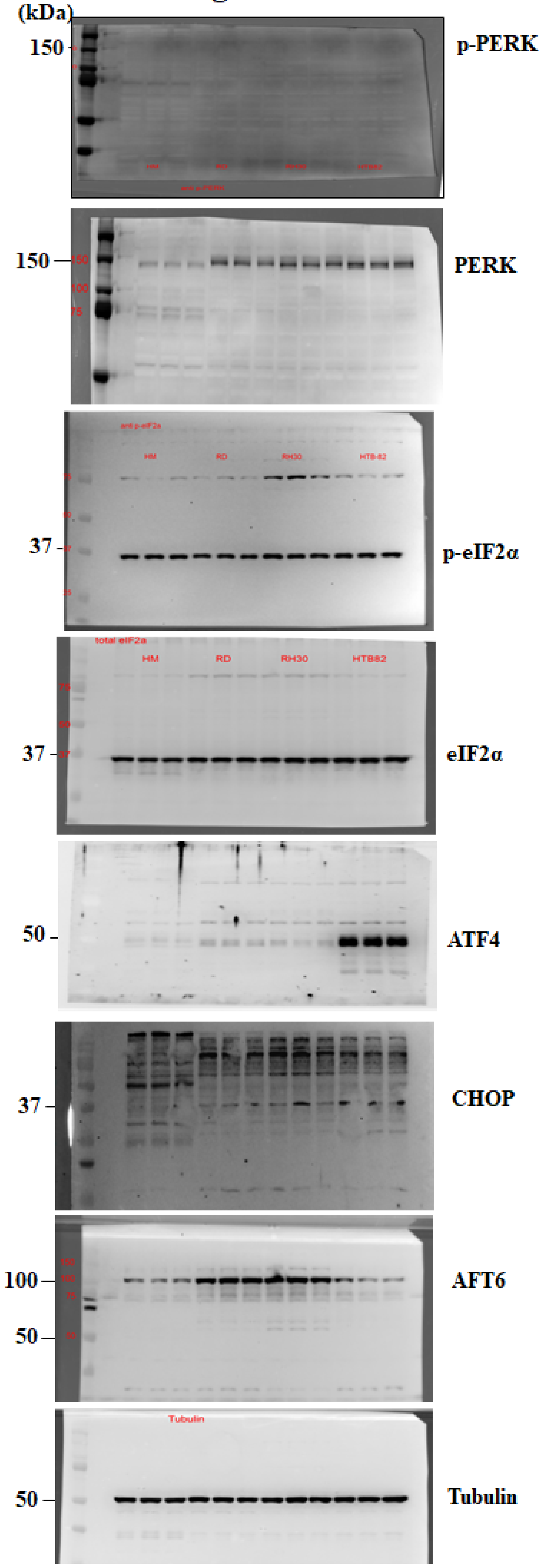

FIGURE S7 (continuation)

Figure S1C

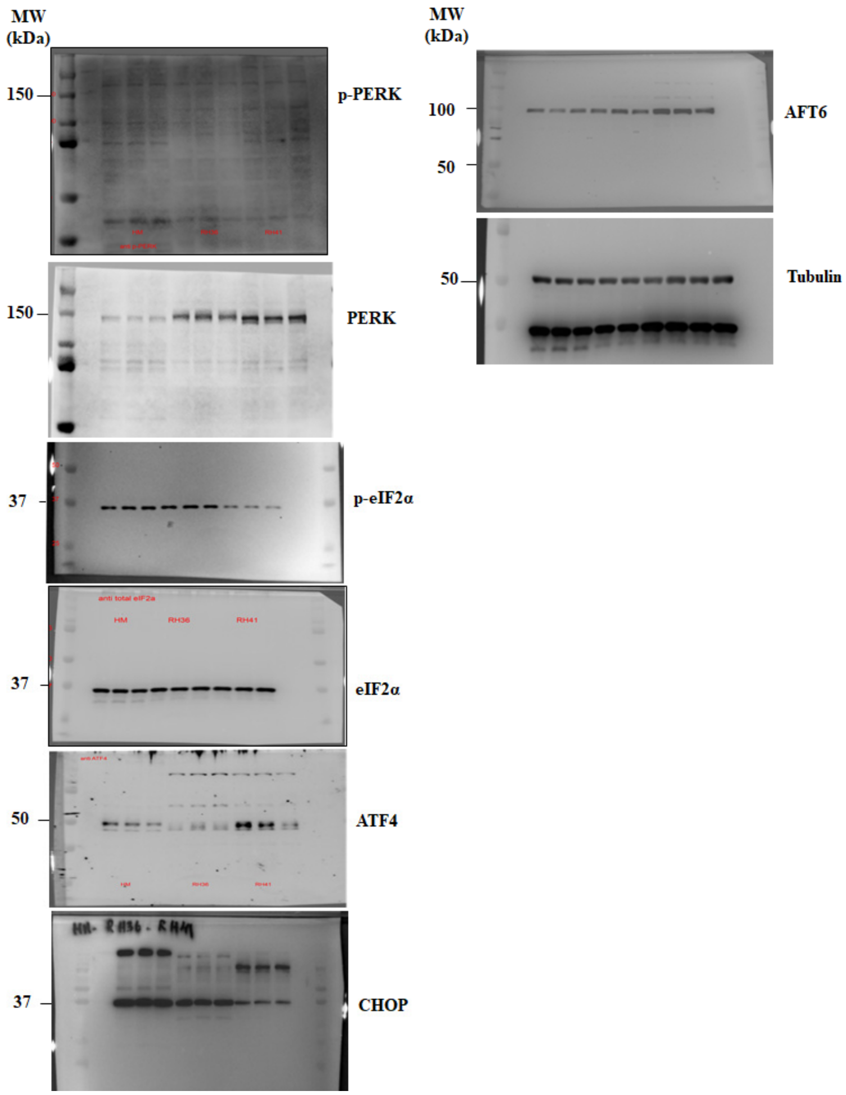

Figure S3C

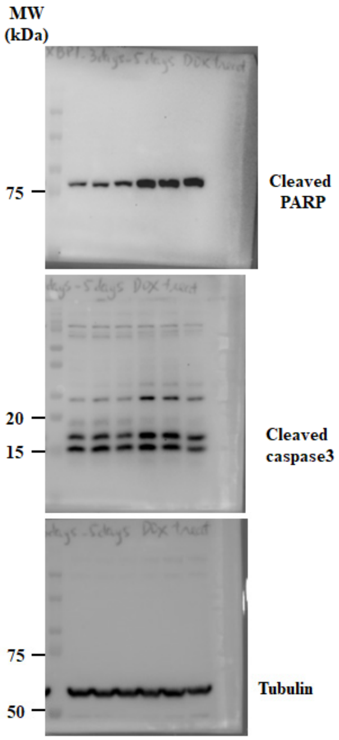

FIGURE S7. Uncropped gel images. Original uncropped images of western blot and agarose gels.

Table S1. List of antibodies, source, and dilution used. ChIP, chromatin immunoprecipitation; IF, immunofluorescence; WB, western blot.

Antibody	Dilution	Source	Identifier
Rabbit-anti-p-ERN1	1:1000 (WB)	Abnova	# PAB12435
Rabbit-anti-IRE1 alpha	1:1000 (WB)	Cell Signaling Technology	# 3294
Rabbit-anti-sXBP1	1:1000 (WB)/1:50 (ChIP)	Cell Signaling Technology	# 40435
Rabbit-anti-alpha Tubulin	1:1000 (WB)	Cell Signaling Technology	# 2144
Rabbit-anti-GAPDH	1:1000 (WB)	Cell Signaling Technology	# 2118
Rabbit-anti-Cleaved PARP	1:1000 (WB)	Cell Signaling Technology	# 5625
Rabbit-anti-Cleaved Caspase 3	1:1000 (WB)	Cell Signaling Technology	# 9661
Rabbit-anti-KLF4	1:1000 (WB)	Cell Signaling Technology	# 12173
Rabbit-anti-Sox9	1:1000 (WB)	Cell Signaling Technology	# 82630
Mouse-anti-Myosin heavy chain	1:500 (WB)/1:50 (IF)	DSHB	# MF20
Mouse-anti-Myogenin	1:500 (WB)/1:50 (IF)	Invitrogen	# MA5-11486
Rabbit-anti-BMPR1A	1:1000 (WB)	Proteintech	#82928-1-RR
Rabbit-anti-p-Smad1	1:1000 (WB)	Cell Signaling Technology	# 5753
Rabbit-anti-Smad1	1:1000 (WB)	Cell Signaling Technology	# 6944
Rabbit-anti-Ki67	1:500 (IF)	Invitrogen	#MA5-14520
Rabbit-anti-XBP1 (For uXBP1)	1:1000 (WB)	Abcam	# ab37152
Rabbit-anti-p-PERK	1:1000 (WB)	Cell Signaling Technology	# 3179S
Rabbit-anti-PERK	1:1000 (WB)	Cell Signaling Technology	# 3192
Mouse-anti-CHOP	1:1000 (WB)	Cell Signaling Technology	# 2895S
Rabbit-anti-p-eIF2a	1:1000 (WB)	Cell Signaling Technology	# 9721
Rabbit-anti-eIF2a	1:1000 (WB)	Cell Signaling Technology	# 9722
Rabbit-anti-ATF4	1:1000 (WB)	Cell Signaling Technology	# 11815
Rabbit-anti-ATF6	1:1000 (WB)	Cell Signaling Technology	# 65880
Anti-rabbit Ig, HRP-linked	1:2000 (WB)	Cell Signaling Technology	# 7074S
Anti-mouse Ig, HRP-linked	1:2000 (WB)	Cell Signaling Technology	# 7076
Anti-Mouse IgG2 AF594	1:1000 (IF)	Invitrogen	# A211135
Anti-Mouse IgG2b AF488	1:1000 (IF)	Invitrogen	# A21141
Anti-Rabbit IgG AF488	1:1000 (IF)	Invitrogen	# A32731

Table S2. Average TPM scores in control RD cultures expressing SCR shRNA.

Gene	Avg TPM value	Gene	Avg TPM value	Gene	Avg TPM value	Gene	Avg TPM value
KLF4	6.019	CAV3	0.111	PTGES	4.079	IFT52	47.469
KLF7	4.842	MYH1	0.185	EDN3	192.454	TRNP1	5.090
LGR5	18.897	MYH4	0.286	VAX1	26.690	EML1	18.746
ALDH1A3	4.151	MYH2	0.117	IGFBP4	5.031	HOXB4	15.241
SOX7	4.034	MYOG	11.005	RAC2	4.628	GNG2	88.089
ZEB1	11.188	MYL9	74.677	CDKN1B	18.663	SERPINF1	58.095
BMI1	17.633	MKX	0.009	IRF2	17.648	FOS	2.901
NGFR	324.698	NPNT	0.704	COL8A1	10.102	PRKCQ	1.741
CD34	7.666	MYL6	1301.628	ZFP36L1	54.502	IFT74	8.142
SOX9	13.691	MEF2B	0.605	USP13	12.948	CEBPA	3.303
ITGA7	13.956	MYL1	8.193	EBI3	0.176	SERPINB1	10.740
MYL4	0.508	ACTC1	58.935	FGF18	0.943	MAB21L1	1.678
TNNC1	0.464	BMPR1A	58.138	CTHRC1	208.887	BTG1	42.452
LAMA1	0.021	BMPR2	12.381	MMP14	52.950	FGF1	2.081
TTN	0.053	SMAD1	40.768	C1QL4	1.618	PIK3CB	4.232
MYOZ2	0.057	SMAD2	41.776	SIX2	90.733	DSN1	38.102
ANKRD1	0.615	SMAD3	14.219	FKBP1B	5.414	CD9	3.895
ANK2	2.921	SMAD4	14.593	VSTM4	16.825	FRS2	3.996
TPM1	58.012	SMAD5	10.406	EGR1	9.129	YPEL5	26.900
TNNI1	12.452	PRDX2	357.515	SIX1	72.489	BTRC	12.350
TNNT2	60.450	TACC2	91.255	GJA1	54.345	LIPA	40.381
TCF21	0.000	ADA	54.406	CAV2	17.855	EDNRA	22.555
MYOD1	85.531	JUNB	19.555	FGF13	60.769	WNT5A	29.052
HDAC9	0.561	COL8A2	0.258	TEK	3.703	MAP2K1	538.223
