# Supplementary material for: Targeting the IRE1α-XBP1 signaling axis impairs tumor growth and promotes myogenic differentiation in rhabdomyosarcoma
Source: Commun Biol. 2026 May 6;9:937. doi: 10.1038/s42003-026-10184-1 (PMC13351075; doi:10.1038/s42003-026-10184-1)
Supplement: Supplementary file 3 — Description of Additional Supplementary Files [file 42003_2026_10184_MOESM3_ESM.pdf]

Description of Additional Supplementary File

File name: Supplementary data

Description: All numerical data for all Figures
