# Supplementary material for: Targeting the IRE1α-XBP1 signaling axis impairs tumor growth and promotes myogenic differentiation in rhabdomyosarcoma
Source: Commun Biol. 2026 May 6;9:937. doi: 10.1038/s42003-026-10184-1 (PMC13351075; doi:10.1038/s42003-026-10184-1)
Supplement: Supplementary file 5 — Reporting Summary [file 42003_2026_10184_MOESM5_ESM.pdf]

nature portfolio

Corresponding author(s): COMMSBIO-25-12270 (Ashok Kumar)

Last updated by author(s): April 20, 2026

Reporting Summary

Nature Portfolio wishes to improve the reproducibility of the work that we publish. This form provides structure for consistency and transparency in reporting. For further information on Nature Portfolio policies, see our Editorial Policies and the Editorial Policy Checklist.

Statistics

For all statistical analyses, confirm that the following items are present in the figure legend, table legend, main text, or Methods section.

n/a	Confirmed	
☐	☒	The exact sample size (*n*) for each experimental group/condition, given as a discrete number and unit of measurement
☐	☒	A statement on whether measurements were taken from distinct samples or whether the same sample was measured repeatedly
☐	☒	The statistical test(s) used AND whether they are one- or two-sided<br>*Only common tests should be described solely by name; describe more complex techniques in the Methods section.*
☒	☐	A description of all covariates tested
☐	☒	A description of any assumptions or corrections, such as tests of normality and adjustment for multiple comparisons
☐	☒	A full description of the statistical parameters including central tendency (e.g. means) or other basic estimates (e.g. regression coefficient) AND variation (e.g. standard deviation) or associated estimates of uncertainty (e.g. confidence intervals)
☒	☐	For null hypothesis testing, the test statistic (e.g. *F*, *t*, *r*) with confidence intervals, effect sizes, degrees of freedom and *P* value noted<br>*Give P values as exact values whenever suitable.*
☒	☐	For Bayesian analysis, information on the choice of priors and Markov chain Monte Carlo settings
☒	☐	For hierarchical and complex designs, identification of the appropriate level for tests and full reporting of outcomes
☒	☐	Estimates of effect sizes (e.g. Cohen's *d*, Pearson's *r*), indicating how they were calculated

Our web collection on statistics for biologists contains articles on many of the points above.

Software and code

Policy information about availability of computer code

Data collection	Data were collected using standard, commercially available software.
Data analysis	Data analysis was performed using standard, commercially available software.

For manuscripts utilizing custom algorithms or software that are central to the research but not yet described in published literature, software must be made available to editors and reviewers. We strongly encourage code deposition in a community repository (e.g. GitHub). See the Nature Portfolio guidelines for submitting code & software for further information.

Data

Policy information about availability of data

All manuscripts must include a data availability statement. This statement should provide the following information, where applicable:

- Accession codes, unique identifiers, or web links for publicly available datasets
- A description of any restrictions on data availability
- For clinical datasets or third party data, please ensure that the statement adheres to our policy

Publicly available gene expression datasets analyzed in this study are available in the Gene Expression Omnibus (GEO) under accession numbers GSE108022 and GSE141690. No restrictions apply to the availability of these data.

Research involving human participants, their data, or biological material

Policy information about studies with human participants or human data. See also policy information about sex, gender (identity/presentation), and sexual orientation and race, ethnicity and racism.

Reporting on sex and gender	This study did not enroll or involve living human participants.
Reporting on race, ethnicity, or other socially relevant groupings	This study did not involve recruitment of human participants.
Population characteristics	No human participants were enrolled
Recruitment	No human participants were recruited for this study
Ethics oversight	This study did not involve direct interaction with human participants

Note that full information on the approval of the study protocol must also be provided in the manuscript.

Field-specific reporting

Please select the one below that is the best fit for your research. If you are not sure, read the appropriate sections before making your selection.

☒ Life sciences ☐ Behavioural & social sciences ☐ Ecological, evolutionary & environmental sciences

For a reference copy of the document with all sections, see nature.com/documents/nr-reporting-summary-flat.pdf

Life sciences study design

All studies must disclose on these points even when the disclosure is negative.

Sample size	The sample sizes (n) are defined as independent biological replicates or individual animals and reported in the figure legends or method.
Data exclusions	No data were excluded from the analyses.
Replication	All key experiments were replicated at least three times using independent biological replicates, similar results were obtained across replicates.
Randomization	For in vivo xenograft experiments, mice were randomized into treatment groups once tumors reached the specified size. Randomization was not applicable to in vitro cell culture experiments.
Blinding	Blinding was not performed during data collection or analysis. Outcome measures were objective and quantitative (cell counts, EdU incorporation, tumor volume measurements), minimizing the risk of bias.

Reporting for specific materials, systems and methods

We require information from authors about some types of materials, experimental systems and methods used in many studies. Here, indicate whether each material, system or method listed is relevant to your study. If you are not sure if a list item applies to your research, read the appropriate section before selecting a response.

Materials & experimental systems

n/a	Involved in the study	
☐	☒	Antibodies
☐	☒	Eukaryotic cell lines
☒	☐	Palaeontology and archaeology
☐	☒	Animals and other organisms
☒	☐	Clinical data
☒	☐	Dual use research of concern
☒	☐	Plants

Methods

n/a	Involved in the study	
☒	☐	ChIP-seq
☐	☒	Flow cytometry
☒	☐	MRI-based neuroimaging

Antibodies

Antibodies used	All antibodies used in this study are listed in Table 1, including target, host species, supplier, and catalog number.

Validation	Antibody specificity was validated by a combination of approaches. For immunoblotting, specificity was assessed by detection of bands at the expected molecular weight and by loss or reduction of signal following genetic knockdown (shRNA-mediated silencing of IRE1α, XBP1, or BMPR1A) or pharmacological inhibition where applicable. Un-cropped, immunoblot images are provided in the FIGURE S5.

Eukaryotic cell lines

Policy information about cell lines and Sex and Gender in Research

Cell line source(s)	RD, RH30, and HTB82 rhabdomyosarcoma cell lines were obtained from the American Type Culture Collection (ATCC). RH36 and RH41 cell lines were kindly provided by the Houghton laboratory (UT Health San Antonio, USA). Primary human skeletal muscle myoblasts were obtained from the Darabi laboratory (University of Houston, USA).
Authentication	Cell lines obtained from ATCC were authenticated by the supplier. Cell lines obtained from collaborating laboratories were used at low passage and were authenticated by the original providers. No additional authentication was performed by the authors.
Mycoplasma contamination	All cell lines were routinely tested and confirmed to be mycoplasma-free using the Universal Mycoplasma Detection Kit (ATCC 30-1012K).
Commonly misidentified lines (See ICLAC register)	None of the cell lines used in this study are listed as commonly misidentified or cross-contaminated in the ICLAC register.

Animals and other research organisms

Policy information about studies involving animals; ARRIVE guidelines recommended for reporting animal research, and Sex and Gender in Research

Laboratory animals	Six-week-old male nude (Nu/Nu) mice were purchased from Charles River Laboratories and used for subcutaneous xenograft experiments.
Wild animals	This study did not involve wild animals.
Reporting on sex	Only male mice were used in this study to maintain consistency with prior xenograft studies and to reduce variability. Sex was not considered as a biological variable.
Field-collected samples	This study did not involve field-collected samples.
Ethics oversight	All animal procedures were approved by the Institutional Animal Care and Use Committee (IACUC) of the University of Houston and were conducted in accordance with institutional and national guidelines for the care and use of laboratory animals.

Note that full information on the approval of the study protocol must also be provided in the manuscript.

Plants

Seed stocks	This study did not use plant material or seed stocks.
Novel plant genotypes	No novel plant genotypes were generated or used in this study.
Authentication	This study did not use plant material or seed stocks. Authentication of plant material was not applicable.

Flow Cytometry

Plots

Confirm that:

☒ The axis labels state the marker and fluorochrome used (e.g. CD4-FITC).

☒ The axis scales are clearly visible. Include numbers along axes only for bottom left plot of group (a 'group' is an analysis of identical markers).

☒ All plots are contour plots with outliers or pseudocolor plots.

☒ A numerical value for number of cells or percentage (with statistics) is provided.

Methodology

Sample preparation	Cells were harvested by trypsinization, washed with phosphate-buffered saline (PBS), and processed according to the manufacturers' instructions for each assay. For EdU incorporation assays, cells were labeled with EdU prior to fixation and permeabilization. For apoptosis assays, cells were stained with Annexin V–FITC and propidium iodide. For surface marker analysis, live cells were stained with fluorophore-conjugated antibodies under non-permeabilizing conditions.
Instrument	Flow cytometry data were acquired using a BD Accuri C6 flow cytometer.
Software	Flow cytometry data were analyzed using FlowJo software.
Cell population abundance	Cell population abundance was quantified as the percentage of cells within the defined gates. Values are presented as mean ± SD from independent biological replicates.
Gating strategy	Gating strategy was applied across all experiments to exclude debris and doublets.

☒ Tick this box to confirm that a figure exemplifying the gating strategy is provided in the Supplementary Information.
