# Supplementary material for: Targeting the IRE1α-XBP1 signaling axis impairs tumor growth and promotes myogenic differentiation in rhabdomyosarcoma
Source: Commun Biol. 2026 May 6;9:937. doi: 10.1038/s42003-026-10184-1 (PMC13351075; doi:10.1038/s42003-026-10184-1)
Supplement: Supplementary file 1 — Transparent Peer Review file [file 42003_2026_10184_MOESM1_ESM.pdf]

nature portfolio

Peer Review File

Targeting the IRE1α-XBP1 signaling axis impairs tumor growth and promotes myogenic differentiation in rhabdomyosarcoma

Corresponding Author: Professor Ashok Kumar

This file contains all reviewer reports in order by version, followed by all author rebuttals in order by version.

Version 0:

Reviewer comments:

Reviewer #1

(Remarks to the Author)

Although the IRE1α/XBP1 branch of the unfolded protein response has been implicated in multiple adult malignancies, its role in rhabdomyosarcoma (RMS) across both embryonal and alveolar subtypes has not previously been investigated. This study shows high gene expression in data from patient tumour specimens and pathway activation in RMS cell lines that leads to BMP-SMAD1 signaling in-keeping with previous reports of BMP-SMAD1 signaling in RMS. Correlative expression data indicates that IRE1α/XBP1 maintains an undifferentiated, stem-like state, promoting proliferation and cancer stem cell properties consistent with the failure of RMS to terminally differentiate. The authors support these features through functional analyses of cell lines (kd and overexpression). Treating with a tool compound targeting IRE1α activity at doses below those expected to produce off-target effects is supportive of therapeutic relevance. However, the relationships at a cellular level between these induced phenotypes, the EMT indicated as well as support and understanding for an enhanced sensitivity to a combination of vincristine/IRE1α tool inhibitor need clarification.
The use of inducible XBP1 knockdown in xenograft models provides additional functional validation and consistent with potential for a new disease-relevant context for this stress-response pathway.
Demonstrating involvement of the IRE1α/XBP1 in RMS could open up a new area in the RMS field and search for new therapeutic options. However, I think the manuscript requires attention to a number of areas.

Comments:
• Fig 1 show high expression in patient samples of RMS relative to skeletal muscle and cell lines relative to human myoblasts. Fig 1 and 2: is the RNA seq in 1B and C for unspliced or spliced XPB1?
• Can IRE1a regulate levels of unspliced XP1 as well as sXBP1? – fig 1D, F. sizes of Western bands are not specified and unspliced XBP1 should be shown too.
• Should mention whether the XBP1 shRNA is targeting spliced or unspliced XPB1?
• If uXBP1 is being expressed in HM, what is the effect of reducing its expression in these cells, and also in these cell after differentiating them, compared with the RMS cell line?
• What is the likely explanation for no effect of kd of IRE1 and XBP1 in RH30 and it is not clear why this is considered as an interesting result on p6.
• Fig 4: The increase in vincristine induced apoptosis by 4u8C seems very small, and the & symbol is not explained. This does not support a “markedly synergistic cytotoxic effect”. The effect of the combination treatment as additive or synergistic should be formally assessed e.g. a combination index, Bliss score. Experiments should evidence how these drugs may work together, vincristine on dividing cells and 4u8C affecting differentiation/stemness/apoptosis (e.g. enhanced apoptosis that is discussed om p12, but unclear with effects of 4u8C promoting differentiation/decreased proliferation as this could reduce effectiveness of vincristine)
• Although levels of stem cell capacity is shown, better understanding, or at least discussion, is required for the process of repressing “stemness” described – is this to do with promoting this population of cells to differentiate? “EMT”. Similarly, there is little explanation of the inhibition of genes expression associated with EMT.

• Fig 6 In Fig 6F has two bands for Myogenin where as other blots show only one, can this be explained?
• Fig 6 and 8. In addition to MyH, Myogenin levels are shown by western blot to increase. In normal myogenesis Myogenin levels tend to increase in myocytes and myotubes but then decreases towards the final stages of terminal differentiation in the myofiber when highest MyH expression occurs. This suggest either cells are transition or cells are at different stages as defined by these markers of differentiation exist. Co-IF for these markers or some further experimental analyses could help better understand this and how the different phenotypes of cell death, stemness and differentiation are related.
• Figure 7A BMPR2 should be investigated in addition to BMPR1A unless there is some clear rationale.
• In vivo testing of 4u8C v kd could have added support towards clinical translation.
• The functional work is reliant on a limited number of cells lines. Use of recently derived in vitro and in vivo models from patient samples are generally expected to better represent the disease.
• Discussion should consider pros and cons of targeting the previously described involvement of the TGF-Beta/Smad2/3 and BMP-driven Smad1/5/8 pathways in RMS versus XBP1 and the IRESα/XBP1.

Minor comments:
• Arrangement of A.B.C. etc in some figures is odd – sometimes consecutively horizontal or sometimes vertical.
• P2 line 1 implies rhabdomyosarcoma (RMS) are the most aggressive pediatric soft tissue sarcoma – not necessarily true. Line 3 whilst the RMS resemble developing muscle their origin in patients is still unclear and experimental data shows that they can arise form other cell types.
• P3 PAX3-FOXO1 and PAX7- current nomenclature should be PAX3::FOXO1. P4 tumor protein 53 (TP53) – protein usually written as p53

Reviewer #2

(Remarks to the Author)
In this manuscript, Kumar and colleagues investigate the role of the IRE1a/XBP1 pathway in the pathogenesis of rhabdomyosarcoma (RMS). This pathway, which acts cooperatively with the other two branches of the unfolded protein response (UPR), is commonly upregulated in many forms of cancer. While the role of IRE1a/XBP1 is known to be context dependent, it is generally believed to function as a survival mechanism that mitigates protein overload.

The authors investigate the relevance of IRE1a/XBP1 signaling in archived data and in a variety of experimental models. In general, these experiments are comprehensive and well presented. The results suggest fairly consistent and statistically significant increases in IRE1a/XBP1 pathway activation in RMS, although in many cases the magnitude of the effects appear to be modest. Perhaps the most definitive experiment is the in vivo tumor growth study shown in figure 9, in which the inducible knockdown of XBP1 clearly inhibits tumor growth. The observation of apoptotic signaling after XBP1 knockdown is consistent with a protective role for the IRE1a/XBP1 pathway. Regarding the pro-proliferative effect of pathway activation, the authors provide a plausible mechanism by directly linking XBP1 to BMPR1A upregulation.

Among the more actionable findings is the potentiation of vincristine by a small molecule inhibitor (4u8C). This in vitro result raises the question of whether a synergistic effect between vincristine and XBP1i could be observed in the RMS tumor growth assay. This experiment may well be beyond the reasonable scope of the current study, but some additional discussion would be warranted.

Reviewer #3

(Remarks to the Author)
Using multiple rhabdomyosarcoma (RMS) cell lines, the authors present evidence showing the IRE1α-XBP axis, a branch of the unfolded protein response (UPR), is a regulator of RMS growth and differentiation. Overall, these findings are intriguing and particularly valuable for readers interested in RMS cell biology. However, in some data, it appeared that IRE1α-XBP inhibition has a relatively weak effect on altering RMS phenotypes, somewhat diminishing the persuasiveness of the interpretation. This manuscript would be strengthened by the following considerations.

1. The authors showed that the IRE1α-XBP axis is activated in RMS tumor samples and cell lines (Fig. 1). However, Figures 1D–G show only the expression levels of sXBP1 in HM and RMS cell lines. Because the XBP1 mRNA levels in Figures 1A and 1B may reflect the total amount of both unspliced and spliced forms, demonstrating the protein expression level of unspliced XBP1 is useful for estimating the intensity of IRE1α-XBP pathway activation. In addition, it will be informative to show whether PERK-ATF4 and ATF6, other UPR pathways, are also activated in RMS.

2. The authors claim that pharmacological inhibition of the IRE1α/XBP1 pathway enhances vincristine-induced cell death in RMS cell lines (Figure 4). Unfortunately, however, vincristine and the IRE1 inhibitor 4μ8C were evaluated at only one dose each. To properly assess synergistic effects, a dose-dependent effect must be demonstrated. Furthermore, the combination of vincristine and 4μ8C slightly enhances apoptosis induction (Figure 4B) while markedly inhibiting cell proliferation (Figures 4C-E). At the very least, the authors should provide some explanation for this discrepancy.

3. In Fig. 6A, shRNAs against IRE1 and XBP1 appear to exert different effects on the mRNA expression levels of muscle differentiation-related molecules. Furthermore, shRNAs against IRE1 and XBP1 only slightly enhanced the expression of myosin heavy chain (MyHC) protein (Figure 6D), whereas the differentiation index was markedly increased by these shRNA treatments (Figures 6B and 6C). The authors should provide some explanation for these phenomena.

4. The data in Figure 7L appeared to suggest that the sXBP protein also binds to the promoter of the RPL30 gene. If this is correct, the authors should provide some explanation in the Results section.

Minor comments:
5. The description in the legend of Figure 8D stating, "RD cells were treated with 0.1μM LDN193189 (a small molecule inhibitor of BMPR1A) or vehicle alone for 72 h followed by fixation of cells and immunostaining for myogenin or MyHC protein. DAPI was used to counterstain nuclei." may be incorrect.

6. The "RMS)" in the second line of the Discussion section is likely a typo.

7. Western blot and transcriptome data are presented for three replicates. The authors should explicitly state whether these are biological replicates or technical replicates.

Version 1:

Reviewer comments:

Reviewer #1

(Remarks to the Author)

I think that the authors have done a good job clarifying many points. My only comments are suggestions related to the abstract to better reflect the results as they now stand: to indicate that the sXBP1 is aberrant; I question whether the suppression of EMT transition as a phenotype is demonstrated (versus gene expression changes consistent with this) and specifying the synergy with vincristine could be included to exemplify the therapeutic potential.

Reviewer #2

(Remarks to the Author)

Reviewer #3

(Remarks to the Author)
This reviewer thinks that the authors have made the necessary revisions to the manuscript.

Open Access This Peer Review File is licensed under a Creative Commons Attribution 4.0 International License, which permits use, sharing, adaptation, distribution and reproduction in any medium or format, as long as you give appropriate credit to the original author(s) and the source, provide a link to the Creative Commons license, and indicate if changes were made.
In cases where reviewers are anonymous, credit should be given to 'Anonymous Referee' and the source.
The images or other third party material in this Peer Review File are included in the article's Creative Commons license, unless indicated otherwise in a credit line to the material. If material is not included in the article's Creative Commons license and your intended use is not permitted by statutory regulation or exceeds the permitted use, you will need to obtain permission directly from the copyright holder.
To view a copy of this license, visit https://creativecommons.org/licenses/by/4.0/

RESPONSE TO REVIEWERS' COMMENTS

We sincerely thank the reviewers for their time and effort in evaluating our manuscript and for their insightful comments, which have greatly helped improve the quality of our study. We have carefully revised the manuscript to address their suggestions. All changes made in response to the reviewers' comments are highlighted in blue. Our detailed responses to each comment are provided below.

REVIWER # 1

Although the IRE1α/XBP1 branch of the unfolded protein response has been implicated in multiple adult malignancies, its role in rhabdomyosarcoma (RMS) across both embryonal and alveolar subtypes has not previously been investigated. This study shows high gene expression in data from patient tumour specimens and pathway activation in RMS cell lines that leads to BMP-SMAD1 signaling in-keeping with previous reports of BMP-SMAD1 signaling in RMS. Correlative expression data indicates that IRE1α/XBP1 maintains an undifferentiated, stem-like state, promoting proliferation and cancer stem cell properties consistent with the failure of RMS to terminally differentiate. The authors support these features through functional analyses of cell lines (kd and overexpression). Treating with a tool compound targeting IRE1α activity at doses below those expected to produce off-target effects is supportive of therapeutic relevance. However, the relationships at a cellular level between these induced phenotypes, the EMT indicated as well as support and understanding for an enhanced sensitivity to a combination of vincristine/IRE1α tool inhibitor need clarification. The use of inducible XBP1 knockdown in xenograft models provides additional functional validation and consistent with potential for a new disease-relevant context for this stress-response pathway. Demonstrating involvement of the IRE1α/XBP1 in RMS could open up a new area in the RMS field and search for new therapeutic options. However, I think the manuscript requires attention to a number of areas.

Comments:

• Fig 1 show high expression in patient samples of RMS relative to skeletal muscle and cell lines relative to human myoblasts. Fig 1 and 2: is the RNA seq in 1B and C for unspliced or spliced XPB1?

RESPONSE: We believe that patient samples may have evaluated both unspliced and spliced XBP1 expression although it is not specifically mentioned in the published dataset. We have now measured unspliced XBP1 (uXBP1) as well. We found that there is no increase in the levels of uXBP1 in RMS cell lines compared with human myoblasts (**Fig. 1D-G**).

• Can IRE1a regulate levels of unspliced XBP1 as well as sXBP1? – fig 1D, F. sizes of Western bands are not specified and unspliced XBP1 should be shown too.

RESPONSE: We have now also performed western blot for unspliced XBP1 (uXBP1) protein which showed no major change compared to spliced XBP1 protein between human myoblasts (HM) and RMS cell lines (**Fig. 1D-G**). Furthermore, our new experiments showed that knockdown of IRE1α does not affect the levels of uXBP1 in RMS cells suggesting that IRE1α only affects sXBP1 protein (**Fig. 2A**)

• Should mention whether the XBP1 shRNA is targeting spliced or unspliced XPB1?
RESPONSE: The XBP1 shRNA we used targets only sXBP1 mRNA. We have now specified this throughout the manuscript.

• If uXBP1 is being expressed in HM, what is the effect of reducing its expression in these cells, and also in these cells after differentiating them, compared with the RMS cell line?
RESPONSE: The uXBP1 protein is expressed in human myoblasts. However, there is no difference in the levels of uXBP1 protein between HM and RMS cell lines. Therefore, we designed our shRNA against the sXBP1 mRNA only. Our new data further demonstrate that knockdown of IRE1α reduces the levels of sXBP1 protein, but not uXBP1 (**Fig. 2A**). Finally, we have performed experiments which showed that pharmacological inhibition of IRE1α activity using 4μ8C or knockdown of XBP1 does not affect the proliferation or differentiation of human myoblasts (Supplemental **Fig. S2E-I**).

• What is the likely explanation for no effect of kd of IRE1 and XBP1 in RH30 and it is not clear why this is considered as an interesting result on p6.
RESPONSE: We have removed the word "Interestingly". We believe that the knockdown of IRE1α or sXBP1 had no significant effect on RH30 cells, because compared to all other RMS cells lines, RH30 cells had minimal increase in the p-IRE1α or sXBP1 protein compared to normal human myoblasts suggesting that the IRE1α/XBP1 may not be driving proliferation of these cells. This is also supported by the fact that overexpression of sXBP1 leads to a more robust increase in the proliferation of RH30 cells as compared to RD cells. This has been explained in the "Results" section on Page # 6 (highlighted text).

• Fig 4: The increase in vincristine induced apoptosis by 4u8C seems very small, and the & symbol is not explained. This does not support a "markedly synergistic cytotoxic effect". The effect of the combination treatment as additive or synergistic should be formally assessed e.g. a combination index, Bliss score. Experiments should evidence how these drugs may work together, vincristine on dividing cells and 4u8C affecting differentiation/stemness/apoptosis (e.g. enhanced apoptosis that is discussed on p12, but unclear with effects of 4u8C promoting differentiation/decreased proliferation as this could reduce effectiveness of vincristine)
RESPONSE: We agree with the reviewer that the effect of the combination of two drugs on RMS cells should be formally assessed. We have now performed systematic experiment in which we measured IC50 values for both the drugs and then studied the effect of varying concentration of vincristine and 4μ8C on representative RMS cell lines. We have also quantitatively assessed combination responses using the Bliss independence model. Our analysis of the cell viability results revealed positive Bliss synergy scores, indicating a synergistic interaction between vincristine and 4μ8C. These new results have been added and explained in the "Results" section (Page #7 and 8; **Fig. 4D, E** and **Supplemental Fig. S4A, B**). The symbol "&" on Fig. 4B and C was explained in the Figure legends. It has now been highlighted.

• Although levels of stem cell capacity is shown, better understanding, or at least discussion, is required for the process of repressing "stemness" described – is this to do with promoting this population of cells to differentiate? "EMT". Similarly, there is little explanation of the inhibition of genes expression associated with EMT.
RESPONSE: We thank the reviewer for this important comment. We agree that the mechanisms

underlying the repression of stemness and EMT-associated gene expression required further clarification. In the “Discussion” section, we have now expanded the text to better explain that inhibition of the IRE1α/XBP1 signaling axis appears to reduce stem-like properties of RMS cells while promoting myogenic differentiation. Please refer to the highlighted text on Page # 13.

• Fig 6 In Fig 6F has two bands for Myogenin whereas other blots show only one, can this be explained?

RESPONSE: Myogenin has been reported to undergo post-translational modifications such as phosphorylation (e.g., at T87), methylation (by PRTM1), and mono-ubiquitinoylation which cause a slower migration in SDS-PAGE, producing a second higher molecular weight band. Because the two bands are very close in molecular weight, their detection can also be influenced by gel percentage, electrophoresis running time, protein expression level, and exposure time during immunoblot development. We have now repeated the western blot for myogenin in Figure 6D which also shows two bands for myogenin similar to Fig. 6F and 6L.

• Fig 6 and 8. In addition to MyH, Myogenin levels are shown by western blot to increase. In normal myogenesis Myogenin levels tend to increase in myocytes and myotubes but then decrease towards the final stages of terminal differentiation in the myofiber when highest MyH expression occurs. This suggests either cells are transitioning or cells are at different stages as defined by these markers of differentiation exist. Co-IF for these markers or some further experimental analyses could help better understand this and how the different phenotypes of cell death, stemness and differentiation are related.

RESPONSE: We agree with the reviewer that Myogenin goes up during myogenic differentiation and then goes down. However, that happens in normal myoblasts when they are incubated in differentiation medium. The experiments in RMS are different. Here, the cells are not synchronized, and we didn’t use differentiation medium in these cells. These are cancerous cells which show some spontaneous differentiation which is further increased by the knockdown of IRE1α/XBP1. We have now performed western blot to measure the levels of myogenin and MyHC on day 3 and day 5 (without changing the medium for 5 days) after transducing the cells with sXBP1 shRNA expressing lentiviral particles. Our results (presented here) demonstrate that the levels of myogenin were reduced whereas levels of MyHC are increased in RD cell on day 5 compared to day 3. These results support the notion that even in RMS cells, the level of myogenin go down as the cells undergo terminal differentiation

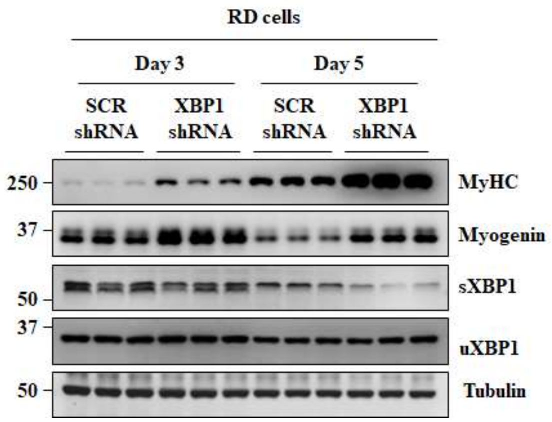

• Figure 7A BMPR2 should be investigated in addition to BMPR1A unless there is some clear rationale.

RESPONSE: In our RNA-Seq dataset, we found that the expression of BMPR2 was not very high. We performed bioinformatics analysis of sXBP1 binding site in the promoter region of various genes related to BMP signaling. We found that sXBP1 binding sites were present only in BMPR1A. Therefore, we pursued only BMPR1A signaling. Moreover, we have now studied the

effect of pharmacological inhibition of BMPR2 in RD cells but could not find any significant effect on their proliferation, survival or differentiation (Page # 11, highlighted text and Supplemental **Fig. S5**).

• In vivo testing of 4u8C v kd could have added support towards clinical translation.
RESPONSE: We had in vivo experiments in our manuscript which showed that pharmacological inhibition of IRE1α using 4μ8C drastically inhibits tumor growth in RD xenograft (**Fig. 9M, N**).

• The functional work is reliant on a limited number of cells lines. Use of recently derived in vitro and in vivo models from patient samples are generally expected to better represent the disease.
RESPONSE: We agree with the reviewer. Due to limited resources, the use of RMS PDX models is outside the scope of this study. However, we will consider performing such experiments in our future studies. We have acknowledged this aspect in the "Discussion" section of the revised manuscript (Page # 14, second last paragraph in the Discussion - highlighted text).

• Discussion should consider pros and cons of targeting the previously described involvement of the TGF-Beta/Smad2/3 and BMP-driven Smad1/5/8 pathways in RMS versus XBP1 and the IREα/XBP1.
RESPONSE: We thank the reviewer for this insightful suggestion. In response, we have expanded the "Discussion" to compare the therapeutic implications of targeting the TGF-β/Smad2/3 signaling pathway and the BMP-Smad1/5/8 signaling pathway with targeting the IRE1α/XBP1 signaling axis in RMS (Please refer to the highlighted first paragraph on Page # 14).

Minor comments:
• Arrangement of A.B.C. etc in some figures is odd – sometimes consecutively horizontal or sometimes vertical.
RESPONSE: We have now arranged various panels in the figures horizontally except in Figure 1 due to the size the panels.

• P2 line 1 implies rhabdomyosarcoma (RMS) are the most aggressive pediatric soft tissue sarcoma – not necessarily true. Line 3 whilst the RMS resemble developing muscles, their origin in patients is still unclear and experimental data shows that they can arise from other cell types.
RESPONSE: We agree with the reviewer. This has been corrected.

• P3 PAX3-FOXO1 and PAX7- current nomenclature should be PAX3::FOXO1. P4 tumor protein 53 (TP53) – protein usually written as p53
RESPONSE: We agree that PAX3::FOXO1 and PAX3::FOXO7 are the recommended modern nomenclature by the HUGO Gene Nomenclature Committee (HGNC). We also agree protein name should be p53. We have made these corrections in the revised manuscript. Thank you!

REVIEWER #2 (Remarks to the Author)

In this manuscript, Kumar and colleagues investigate the role of the IRE1a/XBP1 pathway in the pathogenesis of rhabdomyosarcoma (RMS). This pathway, which acts cooperatively with the other two branches of the unfolded protein response (UPR), is commonly upregulated in many forms of cancer. While the role of IRE1a/XBP1 is known to be context dependent, it is generally believed to function as a survival mechanism that mitigates protein overload.

The authors investigate the relevance of IRE1a/XBP1 signaling in archived data and in a variety of experimental models. In general, these experiments are comprehensive and well presented. The results suggest fairly consistent and statistically significant increases in IRE1a/XBP1 pathway activation in RMS, although in many cases the magnitude of the effects appear to be modest. Perhaps the most definitive experiment is the in vivo tumor growth study shown in figure 9, in which the inducible knockdown of XBP1 clearly inhibits tumor growth. The observation of apoptotic signaling after XBP1 knockdown is consistent with a protective role for the IRE1a/XBP1 pathway. Regarding the pro-proliferative effect of pathway activation, the authors provide a plausible mechanism by directly linking XBP1 to BMPR1A upregulation.

Among the more actionable findings is the potentiation of vincristine by a small molecule inhibitor (4u8C). This in vitro result raises the question of whether a synergistic effect between vincristine and XBP1i could be observed in the RMS tumor growth assay. This experiment may well be beyond the reasonable scope of the current study, but some additional discussion would be warranted.
RESPONSE: We appreciate the highly positive comments by the reviewer. In response to a comment by other two reviewers, we have now performed in vitro experiments studying the synergy of vincristine and 4μ8C in representative ERMS and ARMS cells. We have also now added additional discussion to emphasize that synergistic interaction between vincristine and inhibition of IRE1α/XBP1 axis should be studied in RMS xenograft models (Page # 14, second last paragraph in the Discussion - highlighted text).

REVIEWER #3 (Remarks to the Author):

Using multiple rhabdomyosarcoma (RMS) cell lines, the authors present evidence showing the IRE1α-XBP axis, a branch of the unfolded protein response (UPR), is a regulator of RMS growth and differentiation. Overall, these findings are intriguing and particularly valuable for readers interested in RMS cell biology. However, in some data, it appeared that IRE1α-XBP inhibition has a relatively weak effect on altering RMS phenotypes, somewhat diminishing the persuasiveness of the interpretation. This manuscript would be strengthened by the following considerations.

1. The authors showed that the IRE1α-XBP axis is activated in RMS tumor samples and cell lines (Fig. 1). However, Figures 1D–G show only the expression levels of sXBP1 in HM and RMS cell lines. Because the XBP1 mRNA levels in Figures 1A and 1B may reflect the total amount of both unspliced and spliced forms, demonstrating the protein expression level of unspliced XBP1 is useful for estimating the intensity of IRE1α-XBP pathway activation. In

addition, it will be informative to show whether PERK-ATF4 and ATF6, other UPR pathways, are also activated in RMS.

RESPONSE: We agree with the reviewer. We have now performed additional experiments which showed that unsliced XBP1 (uXBP1) protein levels are comparable between HM and RMS cell lines **(Fig. 1D-G)**. Furthermore, our new experiments demonstrate how the levels of PERK, eIF2α, CHOP, ATF4 and ATF6 protein are affected in RMS cell lines in comparison to human myoblasts (Supplemental **Fig. S1**).

2. The authors claim that pharmacological inhibition of the IRE1α/XBP1 pathway enhances vincristine-induced cell death in RMS cell lines (Figure 4). Unfortunately, however, vincristine and the IRE1 inhibitor 4μ8C were evaluated at only one dose each. To properly assess synergistic effects, a dose-dependent effect must be demonstrated. Furthermore, the combination of vincristine and 4μ8C slightly enhances apoptosis induction (Figure 4B) while markedly inhibiting cell proliferation (Figures 4C-E). At the very least, the authors should provide some explanation for this discrepancy.

RESPONSE: We appreciate reviewer's suggestion. Similar concern was also raised by reviewer # 1. We have now studied the effect of different concentrations of vincristine and 4μ8C on the viability of RMS cell lines. Our analysis of the cell viability results using the Bliss independence model revealed positive Bliss synergy scores, suggesting a synergistic interaction between vincristine and 4μ8C in RMS cell lines (**Fig. 4D, E** and Supplemental **Fig. S4A, B**).

3. In Fig. 6A, shRNAs against IRE1 and XBP1 appear to exert different effects on the mRNA expression levels of muscle differentiation-related molecules. Furthermore, shRNAs against IRE1 and XBP1 only slightly enhanced the expression of myosin heavy chain (MyHC) protein (Figure 6D), whereas the differentiation index was markedly increased by these shRNA treatments (Figures 6B and 6C). The authors should provide some explanation for these phenomena.

RESPONSE: There is a general trend indicating that the expression of most molecules associated with muscle differentiation increases following knockdown of either IRE1α or sXBP1 in RD cells. However, we acknowledge that the magnitude of this increase varies between IRE1α and sXBP1 knockdown conditions. This difference may be explained by the broader functional roles of IRE1α. In addition to mediating the unconventional splicing of XBP1 mRNA to generate the active transcription factor sXBP1, IRE1α regulates cellular responses through regulated IRE1-dependent decay (RIDD) of specific mRNAs and through its kinase activity. The kinase domain of IRE1α can activate signaling pathways such as JNK and potentially NF-κB, which may influence the expression of multiple genes through both direct and indirect mechanisms. Consequently, knockdown of IRE1α may have broader transcriptional effects than knockdown of sXBP1 alone.

We believe that our western blots in **Fig. 6D** during previous submission were not representative. We have now repeated this experiment. Our new results demonstrate appreciable difference in the levels of MyHC in control and IRE1α or sXBP1 knockdown RD cells (new **Fig. 6D, E**).

4. The data in Figure 7L appeared to suggest that the sXBP1 protein also binds to the promoter of the RPL30 gene. If this is correct, the authors should provide some explanation in the Results section.

RESPONSE: RPL30 is commonly used as a positive control in ChIP-qPCR assays, often paired with a Histone H3 antibody to confirm successful immunoprecipitation of DNA. While the functional significance is not known, we have previously reported that there is conserved sXBP1 binding site in the promoter region of RPL30 gene and sXBP1 is enriched at this site (PubMed ID: 41249735).

Minor comments:

5. The description in the legend of Figure 8D stating, "RD cells were treated with 0.1µM LDN193189 (a small molecule inhibitor of BMPR1A) or vehicle alone for 72 h followed by fixation of cells and immunostaining for myogenin or MyHC protein. DAPI was used to counterstain nuclei." may be incorrect.
RESPONSE: This has been corrected.

6. The "RMS)" in the second line of the Discussion section is likely a typo.
RESPONSE: Corrected.

7. Western blot and transcriptome data are presented for three replicates. The authors should explicitly state whether these are biological replicates or technical replicates.
RESPONSE: These are biological replicates. This information has been added to all figure legends.

RESPONSE TO REVIEWERS' COMMENTS

REVIWER # 1

Comment: I think that the authors have done a good job clarifying many points. My only comments are suggestions related to the abstract to better reflect the results as they now stand: to indicate that the sXBP1 is aberrant; I question whether the suppression of EMT transition as a phenotype is demonstrated (versus gene expression changes consistent with this) and specifying the synergy with vincristine could be included to exemplify the therapeutic potential.

RESPONSE: We have now edited the Abstract to better reflect the results as they now stand after revision. EMT transition as a phenotype is demonstrated by measuring invasion and migration assays. We have included vincristine synergy in the Abstract as well. Thank you!

REVIEWER # 2 and # 3

No more comments.
